# Identification of the Active Constituents and Significant Pathways of Shen-qi-Yi-zhu Decoction on Antigastric Cancer: A Network Pharmacology Research and Experimental Validation

**DOI:** 10.1155/2021/6642171

**Published:** 2021-11-22

**Authors:** Shuhong Zeng, Zhibao Yu, Xintian Xu, Yuanjie Liu, Jiepin Li, Danya Zhao, Changjuan Song, Haixia Lu, Yudong Zhao, Weimin Lu, Xi Zou

**Affiliations:** ^1^Affiliated Hospital of Nanjing University of Chinese Medicine, Jiangsu Province Hospital of Chinese Medicine, Nanjing, Jiangsu 210029, China; ^2^No. 1 Clinical Medical College, Nanjing University of Chinese Medicine, Nanjing, Jiangsu 210023, China; ^3^Department of Oncology, Zhangjiagang TCM Hospital Affiliated to Nanjing University of Chinese Medicine, Zhangjiagang, Jiangsu 215600, China; ^4^The First School of Clinical Medicine, Zhejiang Chinese Medicine University, Hangzhou, Zhejiang 310053, China

## Abstract

Shen-qi-Yi-zhu decoction (SQYZD) is an empirical prescription with antigastric cancer (GC) property created by Xu Jing-fan, a National Chinese Medical Master. However, its underlying mechanisms are still unclear. Based on network pharmacology and experimental verification, this study puts forward a systematic method to clarify the anti-GC mechanism of SQYZD. The active ingredients of SQYZD and their potential targets were acquired from the TCMSP database. The target genes related to GC gathered from GeneCards, DisGeNET, OMIM, TTD, and DrugBank databases were imported to establish protein-protein interaction (PPI) networks in GeneMANIA. Cytoscape was used to establish the drug-ingredients-targets-disease network. The hub target genes collected from the SQYZD and GC were parsed via GO and KEGG analysis. Our findings from network pharmacology were successfully validated using an *in vitro* HGC27 cell model experiment. In a word, this study proves that the combination of network pharmacology and *in vitro* experiments is effective in clarifying the potential molecular mechanism of traditional Chinese medicine (TCM).

## 1. Introduction

Gastric cancer (GC) is one of the most common causes of cancer-related mortality worldwide [1]. Currently, the main problems of gastric cancer treatment are drug resistance and complications after surgery, chemotherapy, and radiotherapy [2]. In recent years, great progress has been made in the treatment of tumors by traditional Chinese medicine (TCM) and TCM combined with chemotherapy [3]. Shen-qi-Yi-zhu decoction (SQYZD) was created by National Chinese Medical Master Xu Jing-fan. He is experienced in the diagnosis and treatment of digestive system diseases and has been engaged in TCM clinical practice for more than 60 years.

Several studies have shown that SQYZD is effective in relieving symptoms and improving GC patients' quality of life. A study by Xu found that SQYZD can reduce chemotherapy-induced side effects such as myelosuppression (thrombocytopenia, hemoglobinemia, and leucopenia), gastrointestinal reactions (nausea, vomiting, and diarrhea), and peripheral neurotoxicity [4]. SQYZD can inhibit the transformation, invasion, and metastasis of gastric cancer cells by inhibiting the Wnt/*β*-catenin signaling pathway [5]. Another study by Sun found that SQYZD combined with chemotherapy can improve patients' symptoms such as stomachache, epigastric puffiness, poor appetite, pantothenic acid, loose stool, and fatigue., improve body function, reduce the adverse reactions caused by chemotherapy (nausea, vomiting, and diarrhea), and improve the quality of life of patients after gastric cancer [6]. Therefore, it is necessary to clarify the biological basis and molecular mechanism of the SQYZD. In recent years, with the development of network pharmacology, combined with its characteristics of “Drug-component-target-disease,” it can provide a new idea for the research of disease treatment and drug selection [7].

In this study, a network pharmacology approach was used to explore the potential mechanism of SQYZD in treating GC. Based on some results from network pharmacology, relevant experiments were designed for validation.

## 2. Materials and Methods

### 2.1. Network Pharmacology

#### 2.1.1. Screening of SQYZD for Active Ingredients and Corresponding Targets

SQYZD was composed by the following herbs: Hedysarum Multijugum Maxim (Huang-qi), Codonopsis Radix (Dang-shen), Coicis Semen (Yi-yi-ren), Atractylodes Macrocephala Koidz (Bai-zhu), Curcumae Rhizoma (E-zhu), Poria Cocos (Schw.) Wolf (Fu-ling), Hedyotis Diffusae Herba (Bai-hua-she-she-cao), Agrimonia Eupatoria (Xian-he-cao), and Licorice (Gan-cao).

The chemical constituents of SQYZD were accessed through the Traditional Chinese Medicine Systems Pharmacology database (TCMSP; https://www.tcmsp-e.com/) [8]. The bioactive compounds were retrieved under the retrieval filters of oral bioavailability (OB) ≥ 30% and drug-likeness (DL) ≥ 0.18 [9, 10]. The TCMSP database was also the main source of component-target data. We converted the target names of the bioactive components of SQYZD into gene names with the species limited to “*Homo sapiens*” with the UniProt Knowledgebase (https://www.uniprot.org/) [11].

#### 2.1.2. Identification of Anti-GC Targets

To find the relevant targets comprehensively, information on GC-associated target genes was combined from the following 5 databases: Genecards (https://www.genecards.org), DisGeNET (https://www.disgenet.org/home/), Online Mendelian Inheritance in Man (OMIM; https://omim.org), Therapeutic Target Database (TTD; http://db.idrblab.net/ttd/), and DrugBank (https://www.drugbank.ca) [12–16]. By combining the genes obtained from the 5 databases after removing the duplicates, protein targets associated with GC were identified. The obtained targets were standardized through the UniProt Knowledgebase as mentioned above.

#### 2.1.3. Protein-Protein Interaction (PPI) Network Analysis and Drug-Ingredients-Targets-Disease Network Construction

The overlapping target proteins of GC and SQYZD were used to construct a PPI network with multiple protein patterns on the GeneMANIA platform (http://genemania.org/) [17]. We set the organism type to “Homo sapiens” and left the default settings in place for the other parameters on the Search Tool for the Retrieval of Interacting Genes/Proteins (STRING) platform (https://string-db.org/, version 11.5) [18]. Then, we imported the information on protein interactions into Cytoscape 3.7.2 (Cytoscape Software, Inc., USA) to obtain the drug-ingredients-targets-disease network and perform network analysis [19]. In the network, nodes represent the target proteins, and edges represent the interactions between proteins.

#### 2.1.4. GO and KEGG Pathway Analysis

The Gene Ontology (GO) and Kyoto Encyclopedia of Genes and Genomes (KEGG) pathway enrichment analysis are important methods used to describe the characteristics of candidate targets [20, 21]. We selected the standard *p* value cut off of 0.05 and the *q* value of 0.05 and performed the enrichment analysis with RStudio 3.6.1 (Bioconductor, clusterProfiler) [22].

### 2.2. Experimental Validation

#### 2.2.1. Preparation of SQYZD

All herbs were purchased from Zhongshan Chinese Traditional Medicine Co. (Nanjing, China) and were identified by a Chinese pharmacist. All of the herbs were soaked for 30 min with 1000 ml of double-distilled water, and then, boiled on a medium fire for 30 min, refluxed and extracted. The boiling process was repeated with another 1500 ml of double-distilled water for 30 min. The two extracted solutions were then mixed and further vaporized to 50 ml by boiling. The concentration of raw herbs was 2 g/ml. Sodium bicarbonate was used to correct the pH value of the SQYZD concentration. The extract was stored at −20°C after sterilization and filtered through a 0.22 *μ*m filter.

#### 2.2.2. Mass Spectrometry Analysis

To analyze and identify the chemical constituents in the extracts of SQYZD, we conducted a mass spectrometry analysis. 1 ml of 80% methanol was added to 200 *μ*l SQYZD and vortexed for 5 min. Then, it was centrifuged at 4°C for 10 min at 12000 rpm. The supernatant was taken for ESI-Q-Exactive-Orbitrap MS in positive/negative ion mode (Thermo Fisher, USA). The data collected by high-resolution liquid quality were sorted preliminarily by CD 2.1 (Thermo Fisher, USA) and then retrieved and compared in MzCloud database.

#### 2.2.3. Cell Culture

HGC27 (undifferentiated GC cells) was purchased from the Chinese National Collection of Authenticated Cell Cultures (Shanghai, China). HGC27 was cultured in RPMI-1640 medium (Gibco, USA) with 10% fetal bovine serum (FBS, Every Green, China), 100 U/ml penicillin, and 100 mg/ml streptomycin (Gibco, USA) and was incubated at 37°C in 5% CO_2_.

#### 2.2.4. Cell Counting Kit-8 (CCK-8) Assay

CCK-8 was used to assess the cytotoxicity of SQYZD. After HGC27 cells were seeded in a 96-well culture plate (Corning, USA) at a density of 2 × 10^3^ cells/well for 24 h, various concentrations of SQYZD (0, 1.25, 2.5, 5.0, 7.5, 10, and 12 mg/ml) were used to incubate with the cells. Subsequently, 10 *µ*l CCK-8 solution (Dojindo, Japan) was applied to each well, followed by incubation for 3 h at 37°C in 5% CO_2_. Finally, the absorbance was measured at 450 nm on a microplate reader. The relative cell viability rate was calculated as follows: (treated/control) ×100%. The IC_50_ values were obtained using the logit method.

#### 2.2.5. Colony Formation Assay

We assessed the clonogenic ability of cells using a clone formation assay. 2 × 10^3^ cells/well were seeded in a 6-well plate (Corning, USA). After attachment, the cells were treated with/without different concentrations of SQYZD (0, 2, 4, and 8 mg/ml) and were cultured with a new medium for 14 days after the SQYZD-containing medium was discarded. After fixation with 4% paraformaldehyde for 15 min, the cells were stained with crystal violet solution for 10 min at room temperature. Finally, the plate was washed moderately with water, and the colony with at least 100 cells was counted. Digital photography of colonies was conducted.

#### 2.2.6. TUNEL Staining

The TUNEL assay was used to assess the cell apoptosis. According to the instructions of the manufacturer, DNA fragments were determined using a One Step TUNEL Apoptosis Assay Kit (Beyotime, China). The nucleus was also stained with DAPI (Beyotime, China). TUNEL-negative (blue) and TUNEL-positive (red) cells were observed using a fluorescence microscope (NIKON DS-Qi2, × 200 magnification).

#### 2.2.7. Wound Healing Assay

Cells were inoculated into 6-well plates (Corning, USA), such that the cell density reached about 60%. After cell adhesion, the medium was discarded, and three lines were drawn on the cell monolayer using a sterile 10-*μ*l pipette tip by following the lines present at the bottom of the plate, with three secondary wells for each sample. Scratches were observed after incubations for 12 hours, 24 hours, and 48 hours in 5% CO_2_ at 37°C and were photographed under a microscope (Olympus CKX-41, ×200 magnification).

#### 2.2.8. Transwell Migration Assay

Cell migration analysis was performed using migration chambers. Equal numbers (5 × 10^3^ cell/well) of HGC27 cells in serum-free medium were seeded in the upper chamber (8 *μ*m, 24-well, Corning, USA) and treated with/without different concentrations of SQYZD (0, 2, 4, and 8 mg/ml). A normal medium containing 10% FBS was added to the lower chamber of a 24-well plate as an attractant. The cells were cultured for 24 hours, then cells remaining on the upper surface of the membrane were removed, while cells that migrated to the lower surface of the membrane were fixed in 4% paraformaldehyde for 15 min and stained with crystal violet. Five random fields were photographed under a microscope (Olympus CKX-41, ×200 magnification), and the cell number was counted using Image J software (Image J Software, Inc., USA).

#### 2.2.9. Western Blot Assessment

Whole cell lysates were extracted from HGC27 cells treated with/without different concentrations of SQYZD (0, 2, 4, and 8 mg/ml) and IGF-1 (insulin-like growth factor-1, PEPRO Tech, 5 *μ*M), a growth factor as an agonist to PI3K/AKT/mTOR signaling pathway, using a standard protocol with RIPA lysis buffer (Beyotime, China). The cell lysate samples (10 *μ*g) were electrophoresed in 10% sodium dodecyl-sulfate polyacrylamide gel (SDS-PAGE) and then transferred to PVDF membranes (0.2 *μ*m, Merck Millipore, USA). The membranes were then blocked-in blocking buffer (NCM Biotech, China) for 10 min at room temperature and incubated at 4°C overnight with primary antibodies. After washing three times, the membranes were incubated with HRP-conjugated secondary antibodies. Target/*β*-actin bands were identified with a gel image processing system (ChemiDoc XRS+). Subsequently, relative protein levels were calculated.

#### 2.2.10. Statistical Analysis

Data were analyzed by GraphPad Prism 8.0 (GraphPad Software, Inc., USA) and reported as means ± standard deviation. We used one-way ANOVA followed by Dunnett's t-test to compare between two groups and multiple groups, respectively. Values of *p* < 0.05 were considered statistically significant. All experiments were conducted at least three times.

## 3. Results

### 3.1. Network Pharmacology

#### 3.1.1. Active Ingredients of SQYZD

A total of 787 active ingredients of the nine herb medicines in SQYZD were retrieved from TCMSP, including 87 ingredients in Hedysarum Multijugum Maxim (Huang-qi), 134 ingredients in Codonopsis Radix (Dang-shen), 38 ingredients in Coicis Semen (Yi-yi-ren), 55 ingredients in Atractylodes Macrocephala Koidz (Bai-zhu), 81 ingredients in Curcumae Rhizoma (E-zhu), 34 ingredients in Poria Cocos (Schw.) Wolf. (Fu-ling), 37 ingredients in Hedyotis Diffusae Herba (Bai-hua-she-she-cao), 41 ingredients in Agrimonia Eupatoria (Xian-he-cao), and 280 ingredients in Licorice (Gan-cao). 179 ingredients passed the filters of OB ≥ 30% and DL ≥ 0.18. Finally, 161 active ingredients passed the repetition. The compounds are shown in Supplement 1.

#### 3.1.2. Identification of Candidate Targets of SQYZD for GC Treatment

**Figure 1:**
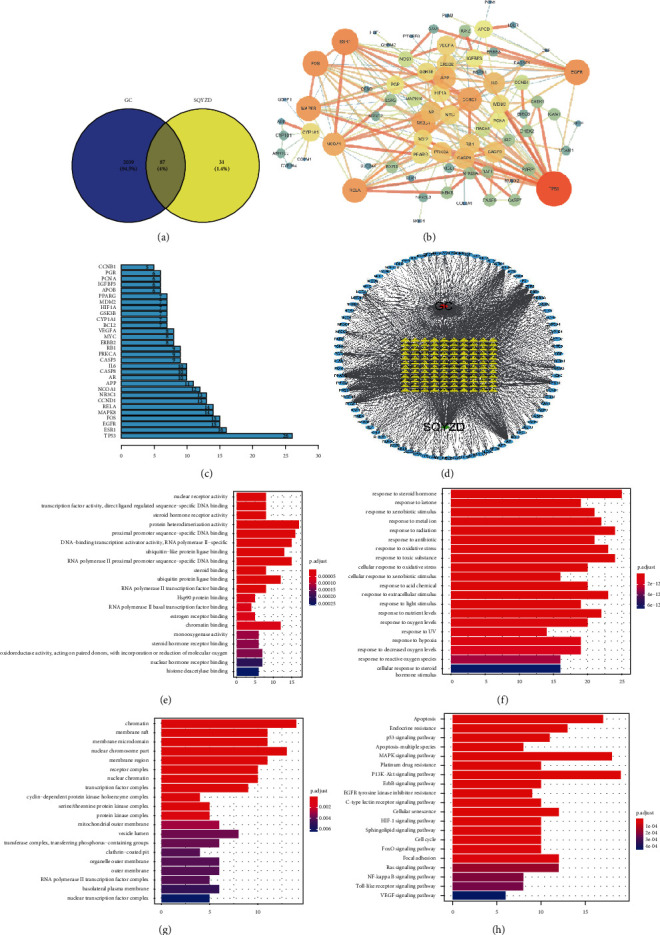
(a) The overlap targets with Venny; (b) the PPI network of targets for SQYZD in treating GC (from dark to light, from small to large, the degrees of freedom increase, and the thicker edges suggest stronger interactions); (c) the top 30 of the core genes; and (d) the SQYZD-GC-related drug targets. The red rectangle, green rectangle, yellow rectangle, and blue rectangle represent disease, herbs, the common targets between SQYZD and GC, and the active ingredient of SQYZD. (e–h) GO and KEGG enrichment analysis and therapeutic targets of SQYZD in treating GC. (e) MF; (f) BP; (g) CC; and (h) KEGG.

After the removal of duplicate targets, a total of 2126 GC-related targets were identified from Genecards, DisGeNET, OMIM, TTD, and DrugBank. The targets were correlated, and a total of 87 targets were indicated as the potential targets with Venny (https://bioinfogp.cnb.csic.es/tools/venny/) (Figure 1(a)).

#### 3.1.3. PPI Network Analysis and Drug-Ingredients-Targets-Disease Network Construction

The 87 common targets were imported to the GeneMANIA database to establish the forecast target PPI network (Figure 1(b)). The confidence level was set to be greater than 0.900, and isolated target proteins were eliminated to obtain protein interaction information. We imported the protein interaction information into Cytoscape3.7.2 to obtain drug-ingredients-targets-disease network analysis. The network had 75 nodes and interacted with 212 edges. We obtained the core genes of the top 30 (Figure 1(c)). The targets of 87 therapeutic targets for the treatment of GC include TP53, ERS1, EGFR, FOS, MAPK8, RELA, CCND1, CASP8, IL-6, CASP3, HIF1A, MDM2, and BCL2. These results demonstrated that these targets were closely related to others in the network and, consequently, might play key roles in GC (Figure 1(d)).

#### 3.1.4. GO Enrichment and KEGG Pathway Analysis

To further illuminate the biological effects involved in the treatment of GC with SQYZD, we performed GO analysis of the 87 GC-related target genes. GO annotation and enrichment of the genes encoding SQYZD were conducted from three aspects: molecular function (MF), biological process (BP), and cellular composition (CC). The KEGG pathway enrichment analysis was also carried out. The most enriched terms with GO and KEGG pathway analysis are shown in Figures 1(e)–1(h).

In detail, the top 10 terms of the GO BP category were mainly enriched in response to steroid hormones, response to radiation, response to toxic substances, response to extracellular stimulus, response to oxidative stress, response to metal ions, response to nutrient levels, response to xenobiotic stimulus, response to antibiotics, and cellular response to oxidative stress. The top 10 terms in the GO CC category were mainly enriched in chromatin, nuclear chromosome part, membrane raft, membrane microdomain, membrane region, receptor complex, nuclear chromatin, transcription factor complex, serine/threonine protein kinase complex, and protein kinase complex. The top 10 terms of the GO MF category were mainly enriched in protein heterodimerization activity, proximal promoter sequence-specific DNA binding, DNA-binding transcription activator activity, RNA polymerase II-specific, RNA polymerase II proximal promoter, sequence-specific DNA binding, ubiquitin-like protein ligase binding, ubiquitin-protein ligase binding, chromatin binding, nuclear receptor activity, transcription factor activity, direct ligand regulated sequence-specific DNA binding, and steroid hormone receptor activity.

According to the *p* value, the PI3K-AKT signaling pathway, the MAPK signaling pathway, apoptosis, endocrine resistance, cellular senescence, the P53 signaling pathway, platinum drug resistance, the ErbB(Her) signaling pathway, the HIF-1 signaling pathway, and the FoxO signaling pathway were within the top 10 pathways.

### 3.2. *In Vitro* Experiments

#### 3.2.1. Mass Spectrometry Result of SQYZD

**Figure 2:**
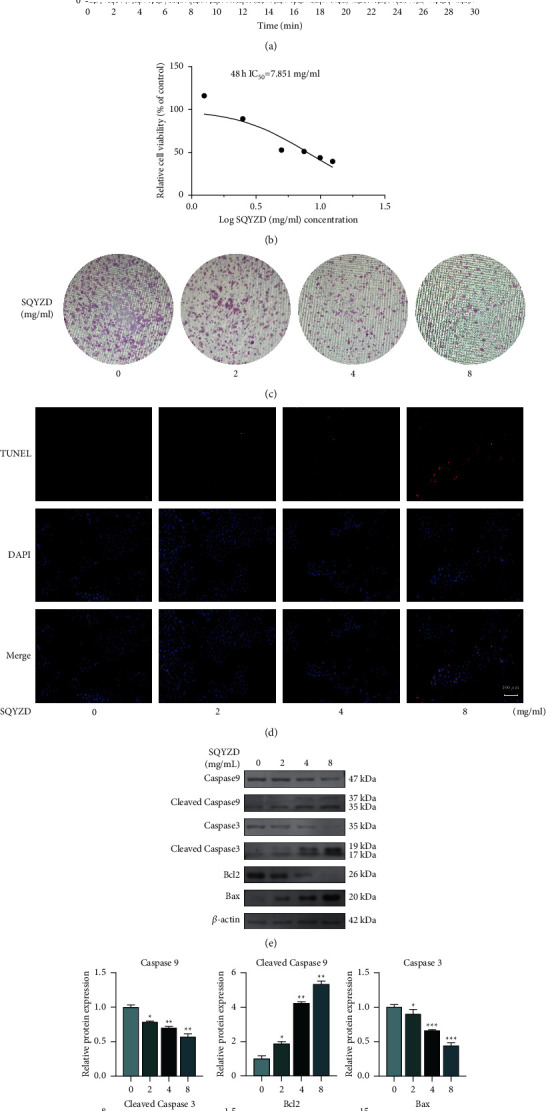
(a) The results of ESI-Q-Exactive-Orbitrap MS in positive/negative ion mode; (b) relative cell viability of HGC27 cells treated with/without SQYZD (0, 2, 4, and 8 mg/ml); (c) colony formation capacity of the HGC27 cells treated with/without SQYZD (0, 2, 4, and 8 mg/ml); (d) apoptosis assessed using a TUNEL assay (TUNEL-positive cells indicated in red) (magnification, ×200); (e) and (f) Expression of apoptosis-related proteins examined using western blots after treatment with/without SQYZD (0, 2, 4, and 8 mg/ml).

The results of ESI-Q-Exactive-Orbitrap MS in positive/negative ion mode are shown in Figure 2(a). A total of 258 compounds were matched in mzCloud for the SQYZD sample. 63 compounds with best match score were over 70 in mzCloud (Supplement 2). The 32 compounds were both in the results of the network pharmacology and the ESI-Q-Exactive-Orbitrap MS.

#### 3.2.2. SQYZD Inhibits the Growth of HGC27 and Induces Apoptosis

SQYZD significantly inhibited the cell proliferation of HGC27. After intervention with SQYZD for 48 h, the IC_50_ was 7.851 mg/ml (Figure 2(b)). Besides, the number of colonies formed by HGC27 decreased with an increase of SQYZD concentration (Figure 2(c)).

With the increase of SQYZD concentration, the TUNEL-positive cells increased gradually (Figure 2(d)). Western blotting of classical apoptotic molecule also revealed that with the increase of SQYZD concentration, the degree of apoptosis of HGC27 cells increased (*p* < 0.05) (Figures 2(e) and 2(f)). The degree of apoptosis in all treatment groups was higher than that in the control group, and it was concentration-dependent. The results of *in vitro* experiments were consistent with the conclusions of network pharmacology.

#### 3.2.3. SQYZD Suppresses the Epithelial-Mesenchymal Transition (EMT) of HGC27

**Figure 3:**
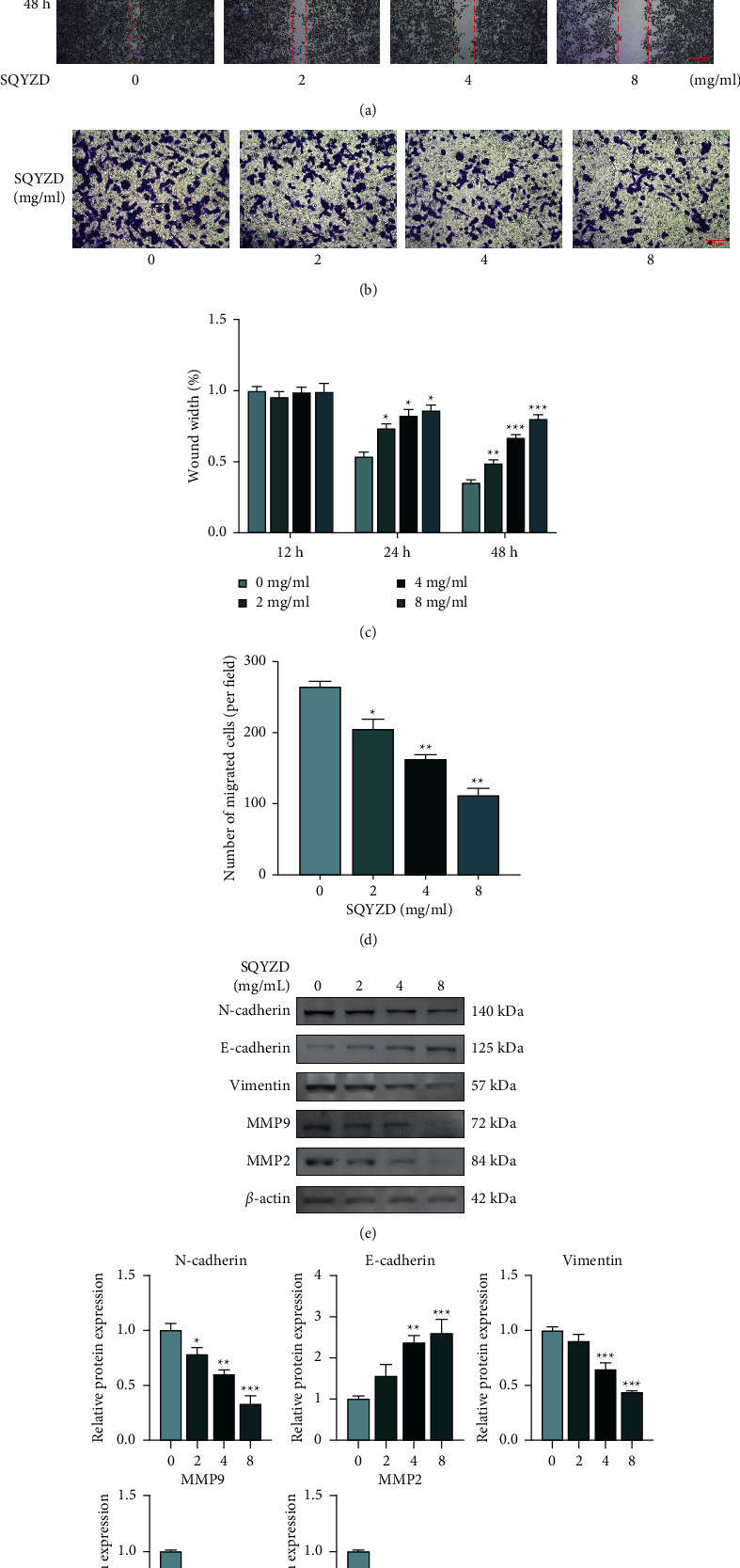
(a) Migratory ability of different groups of HGC27 cells treated with/without SQYZD (0, 2, 4, and 8 mg/ml) examined using wound healing assays. (c) The percentages of wound width at 12 h, 24 h, and 48 h. (b) The migration ability of GC cells after treatment with/without SQYZD (0, 2, 4, and 8 mg/ml). (d) The relative migratory cell number. (e, f) Expression of EMT-related proteins examined using western blots after treatment with/without SQYZD (0, 2, 4, and 8 mg/ml).

An increase in SQYZD concentration significantly inhibited cell wound healing (Figures 3(a) and 3(c)) and migration (Figures 3(b) and 3(d)). Moreover, an increase of SQYZD concentration induced E-cadherin while inhibited N-cadherin, vimentin, and MMP2 and MMP9 levels (*p* < 0.05) (Figures 3(e) and 3(f)). These data indicated that SQYZD inhibited EMT in GC.

#### 3.2.4. SQYZD Inhibits PI3K/AKT/mTOR Signaling in GC

**Figure 4:**
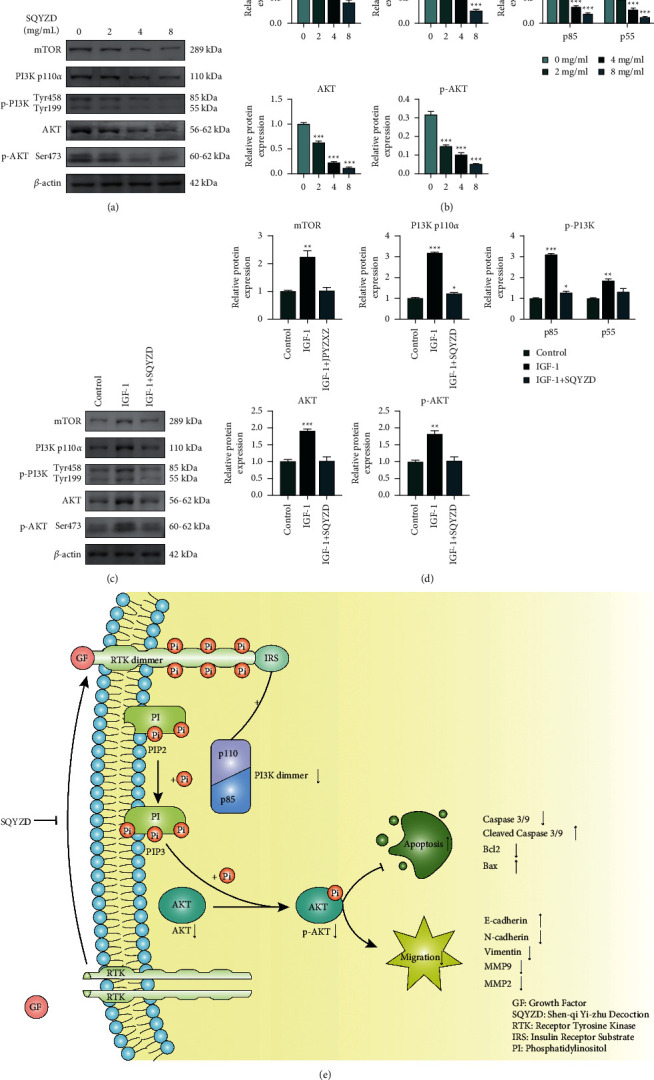
(a) Expression of PI3K/AKT/mTOR-related proteins examined using western blots after treatment with/without SQYZD (0, 2, 4, and 8 mg/ml). (b) Expression of PI3K/AKT/mTOR-related proteins examined using western blots after treatment with 5 *μ*M IGF, an agonist to PI3K/AKT/mTOR signaling pathway, or along with 4 mg/ml SQYZD. (c) Simple mode diagram of SQYZD acting on the PI3K/AKT/mTOR signaling pathway.

To further explore the potential mechanisms of anti-GC, according to the results of KEGG pathway analysis in network pharmacology, we first analyzed the effect of SQYZD on the expression of key molecules in the PI3K/AKT/mTOR axis. Increase of SQYZD concentration inhibited mTOR, PI3K, p-PI3K, AKT, and p-AKT levels (*p* < 0.05) (Figures 4(a) and 4(b)).

In order to further confirm our idea, we conducted verification experiments with IGF-1 (a growth factor as the agonist of PI3K/AKT/mTOR). We observed that mTOR, PI3K, p-PI3K, AKT, and p-AKT levels were significantly in the IGF-1 group (*p* < 0.05). After treating with SQYZD, mTOR, PI3K, p-PI3K, AKT, and p-AKT levels were downregulated in HGC27 cells (Figures 4(c) and 4(d)).

These data indicated that SQYZD suppressed proliferation, antiapoptosis, and EMT might be through inhibiting the PI3K/AKT/mTOR signaling pathway *in vitro*.

## 4. Discussion

In recent years, TCM has played a unique role in the antitumor area. TCM can reduce the toxic and side effects of chemotherapy and improve the quality of GC patients' lives [23, 24]. However, due to the complex chemical composition and unclear pharmacological mechanism of action of TCMs, it is a great challenge to their pharmacological research. The development of network pharmacology provides databases and disease target prediction for TCM research, facilitates the study of active ingredients and mechanisms of TCM, and provides new ideas and methods for the development of TCM modernization.

In our study, the preliminary analysis based on network pharmacology provided a basis for subsequent studies on the pharmacodynamic ingredients and mechanisms of SQYZD. In this study, the active components of SQYZD and their molecular mechanisms in GC were predicted overall, and a variety of potential targets were explored. Meanwhile, we also identified the components in the SQYZD extract by mass spectrometry to verify the rationality of the network pharmacology results.

An approach to combine network pharmacology and experimental verification was used to predict and verify the anti-GC mechanisms of SQYZD in this study. According to the results of network pharmacology, we verified the phenotype of SQYZD in promoting apoptosis and inhibiting EMT and showed its concentration-dependent features by western blot.

The PI3K/AKT/mTOR signaling pathway is an important signaling pathway in cancer, which is active in most cancers and plays an important role in cancer biology. Studies have shown that the PI3K/AKT/mTOR pathway is involved in regulating the occurrence and development of gastric cancer mediated by cell proliferation, antiapoptosis, migration, and glucose metabolism [25, 26]. According to our network pharmacology speculation, SQYZD also plays an antitumor role by inhibiting the PI3K/AKT/mTOR pathway. Through the rescue experiments, we verified that SQYZD can suppress antiapoptosis and EMT by inhibiting the PI3K/AKT/mTOR pathway (Figure 4(e)).

However, there are still some shortcomings in this study. The current work lacks *in vivo* experiments, which will be further verified in the follow-up research.

## Data Availability

The data used to support the findings of this study are available from the corresponding author upon request.

## Supplementary Materials

Supplementary Materials Supplement 1: 161 active ingredients passed the repetition, the compounds are shown in Supplement 1. Supplement 2: 63 compounds of best match score were over 70 in mzCloud are shown in Supplement 2.
