# Supplementary material for: Identification of the Active Constituents and Significant Pathways of Shen-qi-Yi-zhu Decoction on Antigastric Cancer: A Network Pharmacology Research and Experimental Validation
Source: Evid Based Complement Alternat Med. 2021 Nov 22;2021:6642171. doi: 10.1155/2021/6642171 (PMC8629626; doi:10.1155/2021/6642171)
Supplement: Supplementary Materials — Supplement 1: 161 active ingredients passed the repetition, the compounds are shown in Supplement 1. Supplement 2: 63 compounds of best match score were over 70 in mzCloud are shown in Supplement 2. [file 6642171.f1.zip › 6642171.f1/Supplement 2.pdf]

Name	Formula	Annotation Source: Predicted Compositions	Annotation Source: mzCloud Search	Annotation Source: mzVault Search
(+/-)9,10-dihydrox	C18 H34 O4	Full match	Full match	No results
Isoliquiritigenin	C15 H12 O4	Full match	Full match	Full match
Azelaic acid	C9 H16 O4	Full match	Full match	Full match
Gluconic acid	C6 H12 O7	Full match	Full match	Full match
3-Phenyllactic acic	C9 H10 O3	Full match	Full match	Full match
Formononetin	C16 H12 O4	Full match	Full match	Full match
L-Histidine	C6 H9 N3 O2	No results	Invalid mass	Invalid mass
7-Hydroxy-2-(4-hy	C21 H22 O10	Not the top hit	Full match	No results
Corchorifatty acid	C18 H32 O5	Not the top hit	Full match	No results
D-(-)-Quinic acid	C7 H12 O6	No match	Invalid mass	Invalid mass
Isocitric acid	C6 H8 O7	Full match	Not the top hit	Partial match
Trigonelline	C7 H7 N O2	Full match	Full match	Full match
L-Tyrosine	C9 H11 N O3	No match	Invalid mass	Invalid mass
Citric acid	C6 H8 O7	Full match	Full match	Full match
Cyclo(leucylprolyl)	C11 H18 N2 O2	Full match	Full match	No results
Bis(2-ethylhexyl) p	C24 H38 O4	Not the top hit	Full match	Full match
2-[3,8-Dihydroxy-8	C21 H36 O10	Not the top hit	Full match	No results
L-Phenylalanine	C9 H11 N O2	Full match	Full match	Full match
Luteolin	C15 H10 O6	Full match	Full match	No results
4,5-Dicaffeoylquin	C25 H24 O12	Not the top hit	Full match	No results
Ononin	C22 H22 O9	Full match	Full match	No results
Stearamide	C18 H37 N O	Full match	Full match	No results
Chlorogenic acid	C16 H18 O9	Not the top hit	Full match	Full match
Neochlorogenic ac	C16 H18 O9	Not the top hit	Full match	Full match
Hispidulin	C16 H12 O6	Not the top hit	Full match	No results
Vanillin	C8 H8 O3	Full match	Full match	Full match
2,3,4,9-Tetrahydro	C12 H12 N2 O2	Full match	Full match	No results
Dibutyl phthalate	C16 H22 O4	Full match	Full match	Not the top hit
Cyclo(phenylalany	C14 H16 N2 O2	Full match	Full match	No results
Suberic acid	C8 H14 O4	Full match	Full match	Full match
trans-3-Indoleacry	C11 H9 N O2	Full match	Full match	Not the top hit
7-Methyl-3-methyl	C15 H20 O3	No match	Invalid mass	No results
9S,13R-12-Oxophy	C18 H28 O3	Full match	Full match	No results
7-Methyl-3-methyl	C15 H20 O3	No match	Invalid mass	No results
Scoparone	C11 H10 O4	Full match	Full match	Invalid mass
Formononetin	C16 H12 O4	Full match	Full match	Full match
L-Glutamic acid	C5 H9 N O4	Full match	Full match	Full match
Methyl cinnamate	C10 H10 O2	Full match	Full match	No results
9-Oxo-10(E),12(E)-(	C18 H30 O3	Full match	Full match	Full match
4-Ethoxy ethylbenz	C11 H14 O3	Full match	Full match	No results
Bis(2-ethylhexyl)ac	C22 H42 O4	Full match	Full match	Full match
Bis(2-ethylhexyl) se	C26 H50 O4	Full match	Full match	Full match
Guanine	C5 H5 N5 O	No match	Invalid mass	Invalid mass
Hexadecanamide	C16 H33 N O	Full match	Full match	Full match
(+/-)12(13)-DiHOM	C18 H34 O4	No match	Invalid mass	No results
DL-Arginine	C6 H14 N4 O2	No match	Invalid mass	Invalid mass
9-Oxo-ODE	C18 H30 O3	Full match	Full match	Full match

L-(+)-Arginine	C6 H14 N4 O2	Full match	Full match	Full match
(3aR,4aS,5R,7aS,8S	C15 H20 O4	Full match	Full match	Partial match
D-(+)-Glucose	C6 H12 O6	No match	Invalid mass	Invalid mass
Nicotinic acid	C6 H5 N O2	Full match	Full match	Full match
Andrographolide	C20 H30 O5	No match	Invalid mass	No results
Argininosuccinic a	C10 H18 N4 O6	Full match	Full match	No results
(+/-)13-HODE	C18 H32 O3	Full match	Full match	No results
7,8-Bis(hydroxyme	C15 H22 O3	Full match	Full match	No results
(+/-)12(13)-DiHOM	C18 H34 O4	No match	Invalid mass	No results
7-Methyl-3-methyl	C15 H20 O3	Full match	Full match	No results
9S,13R-12-Oxophy	C18 H28 O3	Full match	Full match	No results
α-Eleostearic acid	C18 H30 O2	Full match	Full match	No results
(15Z)-9,12,13-Trihy	C18 H34 O5	Full match	Full match	No results
Nootkatone	C15 H22 O	Full match	Full match	Full match
Uridine	C9 H12 N2 O6	Full match	Full match	Full match
Asparagine	C4 H8 N2 O3	No match	Invalid mass	Invalid mass
3-tert-Butyladipic a	C10 H18 O4	Full match	Full match	Full match
N-Acetyl-DL-trypto	C13 H14 N2 O3	Full match	Full match	Full match
Phenacetin	C10 H13 N O2	Full match	Full match	No results
N-Acetyl-DL-trypto	C13 H14 N2 O3	Full match	Full match	Full match
3-Fluoro-N'-hydrox	C8 H9 F N2 O	No results	Invalid mass	No results
1,9b-Dihydroxy-6,6	C15 H22 O4	No match	Invalid mass	No results
Butyl 4-aminobenz	C11 H15 N O2	Full match	Full match	Full match
1,9b-Dihydroxy-6,6	C15 H22 O4	No match	Invalid mass	No results
Hispidulin	C16 H12 O6	Not the top hit	Full match	No results
DL-Tryptophan	C11 H12 N2 O2	Full match	Full match	Full match
3-Phenyllactic acid	C9 H10 O3	Full match	Full match	Not the top hit
4,5-Dicaffeoylquin	C25 H24 O12	Not the top hit	Full match	No results
2-[3,8-Dihydroxy-8	C21 H36 O10	No match	Invalid mass	No results
2-[3,8-Dihydroxy-8	C21 H36 O10	No match	Invalid mass	No results
Bisphenol B	C16 H18 O2	No match	Invalid mass	No results
7-Methyl-3-methyl	C15 H20 O3	No match	Invalid mass	No results
2-(4-Methyl-3-cycl	C22 H38 O10	Not the top hit	Full match	No results
Sulfamethoxazole l	C10 H11 N3 O4 S	No match	Invalid mass	No results
4-{[3-(Diethylamin	C11 H20 N2 O3	No match	Invalid mass	No results
Bisphenol B	C16 H18 O2	No match	Invalid mass	No results
2-Benzyl-5-[(3S)-1-(	C19 H25 N3 O	No match	Invalid mass	No results
Nikethamide 1-oxi	C10 H14 N2 O2	Full match	Full match	No results
Phenylbutazone	C19 H20 N2 O2	No match	Invalid mass	No results
1,9b-Dihydroxy-6,6	C15 H22 O4	Full match	Full match	No results
Cuminaldehyde	C10 H12 O	Full match	Full match	No results
α,α-Trehalose	C12 H22 O11	Not the top hit	Full match	Full match
3-Methylxanthine	C6 H6 N4 O2	No match	Invalid mass	No results
N-(4-{(R)-Hydroxy[(	C22 H26 N2 O4	No match	Invalid mass	No results
2-(6-Hydroxyhexyl	C11 H18 O5	Full match	Full match	No results
Betaxolol	C18 H29 N O3	No match	Invalid mass	No results
6,18,19-Trihydroxy	C20 H30 O4	No match	Invalid mass	No results
D-(-)-Quinic acid	C7 H12 O6	Full match	Full match	Full match
3-tert-Butyladipic a	C10 H18 O4	Full match	Full match	No results
Norfentanyl	C14 H20 N2 O	Full match	Full match	No results
Indole-3-lactic acid	C11 H11 N O3	Full match	Full match	Not the top hit
Caffeic acid	C9 H8 O4	Full match	Full match	Full match

3-(2,6-Dioxocycloh	C9 H11 N O2	Full match	Full match	No results
N-(1-Adamantyl)-N	C19 H26 N2 O S	No match	Invalid mass	No results
all-cis-4,7,10,13,1	C22 H34 O2	No match	Invalid mass	No results
1-(3-Trifluorometh	C11 H13 F3 N2	No match	Invalid mass	No results
Matairesinol	C20 H22 O6	No match	Invalid mass	No results
1-(4,7-Dimethylpy	C9 H10 N4 O	No match	Invalid mass	No results
3-Isopropylmalic a	C7 H12 O5	Full match	Full match	Not the top hit
Boldenone undecy	C30 H44 O3	Full match	Full match	No results
3,5-Dimethoxyben	C9 H10 O4	Full match	Full match	Partial match
trans-Aconitic acid	C6 H6 O6	Full match	Full match	Full match
{(2R,4S,5R)-5-[3-(3,	C27 H32 N4 O4	No match	Invalid mass	No results
3-[2-(1,3-Benzodio	C25 H28 O11	No match	Invalid mass	No results
Acetophenazine	C23 H29 N3 O2 S	No match	Invalid mass	No results
N-({(2R,4S,5R)-5-[2	C22 H28 N4 O S	No match	Invalid mass	No results
(9Z,12Z)-6,8-Dihyd	C18 H32 O4	Full match	Full match	No results
(3R,5R)-1,3,5-Trihy	C17 H20 O9	Not the top hit	Full match	No results
4,5-Dicaffeoylquin	C25 H24 O12	No match	Invalid mass	No results
N-(3,4-Dimethylph	C17 H15 N3 O	No match	Invalid mass	No results
Fraxetin	C10 H8 O5	Not the top hit	Full match	No results
3',4'-Dihydroxyphe	C9 H10 O3	Full match	Full match	No results
Nicotinamide	C6 H6 N2 O	Full match	Full match	Full match
Hexamethylene dii	C8 H12 N2 O2	No match	Invalid mass	No results
6-(4-Chloro-2-cyclo	C17 H19 Cl N2 O	No match	Invalid mass	No results
α-Eleostearic acid	C18 H30 O2	Full match	Full match	No results
3',4'-Dihydroxyphe	C9 H10 O3	Full match	Full match	No results
1-{4-[(2S,4R)-4-Hyd	C20 H23 F3 N4 O4	No match	Invalid mass	No results
Phenacetin	C10 H13 N O2	Full match	Full match	Full match
1-(1-Hydroxybutyl	C12 H20 O6	Not the top hit	Full match	No results
L-Phenylalanine	C9 H11 N O2	Full match	Full match	Full match
3-(1-hydroxyethyl)	C9 H14 N2 O3	Full match	Full match	No results
(9Z,12Z)-6,8-Dihyd	C18 H32 O4	Full match	Full match	No results
D-Glucosamine	C6 H13 N O5	Full match	Full match	Full match
Cortisol	C21 H30 O5	No match	Invalid mass	No results
4-Methoxycinnam	C10 H10 O3	Full match	Full match	No results
Tropicamide	C17 H20 N2 O2	No match	Invalid mass	No results
N1-{4-[4-(1H-Pyrro	C22 H22 Cl N3 O	No match	Invalid mass	No results
D-(+)-Maltose	C12 H22 O11	No match	Invalid mass	Invalid mass
3-[2-(1,3-Benzodio	C25 H28 O11	No match	Invalid mass	No results
Alprazolam	C17 H13 Cl N4	No match	Invalid mass	No results
5-Fluoro ADB	C20 H28 F N3 O3	No match	Invalid mass	No results
D-(+)-Glucose	C6 H12 O6	No match	Invalid mass	Invalid mass
N4-Acetylsulfamet	C12 H13 N3 O4 S	No match	Invalid mass	No results
Oxyphenbutazone	C19 H20 N2 O3	No match	Invalid mass	No results
5-Fluoro ADB	C20 H28 F N3 O3	No match	Invalid mass	No results
4-Hydroxybenzalde	C7 H6 O2	Full match	Full match	Full match
PEG n8	C16 H34 O9	Not the top hit	Full match	No results
Cuminaldehyde	C10 H12 O	Full match	Full match	No results
Octyl gallate	C15 H22 O5	Full match	Full match	No results
Boldenone undecy	C30 H44 O3	No match	Invalid mass	No results
1,3-Dihydroxy-1-(7	C20 H26 O7	No match	Invalid mass	No results
5-Chloro THJ 018	C23 H21 Cl N2 O	No match	Invalid mass	No match
6 β-Hydroxycortisc	C21 H30 O6	No match	Invalid mass	No results

N-{(1R,9S)-11-[(1-M	C27 H27 N5 O2	No match	Invalid mass	No results
1-(3-Trifluorometh	C11 H13 F3 N2	No match	Invalid mass	No results
3-Hydroxy-3,5,5-tr	C19 H30 O8	No match	Invalid mass	No results
Sorbicillin	C14 H16 O3	Full match	Full match	No results
5-Fluoro AMB meta	C18 H24 F N3 O3	Invalid mass	Invalid mass	No results
1-(3-Trifluorometh	C11 H13 F3 N2	No match	Invalid mass	No results
MN-18 N-(5-hydrox	C23 H23 N3 O2	No match	Invalid mass	No results
5-Isoxazol-5-yl-N-[4	C19 H22 N4 O3 S2	Not the top hit	Full match	No results
Octyl gallate	C15 H22 O5	Full match	Full match	No results
1,9b-Dihydroxy-6,6	C15 H22 O4	Full match	Full match	No results
4-(2,7-Dihydroxy-6	C15 H22 O5	Full match	Full match	No results
Dodecyl sulfate	C12 H26 O4 S	Not the top hit	Full match	Full match
(3R,4S)-4,6,8-Trihy	C11 H12 O6	Full match	Full match	No results
Porphobilinogen	C10 H14 N2 O4	Full match	Full match	No results
7-Hydroxycoumari	C9 H6 O3	Full match	Full match	No results
N4-(2-Methoxyphe	C13 H10 N4 O4	Full match	Full match	No results
(5-Amino[1,2,3]tria	C14 H14 N6 O2	Not the top hit	Full match	No results
N-[(1S,4S)-4-{3,5-D	C23 H21 F3 N4 O	No match	Invalid mass	No results
6-Methoxyquinolin	C10 H9 N O	Full match	Full match	No results
Rutarin	C20 H24 O10	No match	Invalid mass	No match
D-(+)-Malic acid	C4 H6 O5	No results	Invalid mass	Invalid mass
BMK methyl glycid	C11 H12 O3	Full match	Full match	No results
6-Hydroxycaproic a	C6 H12 O3	No results	Invalid mass	Invalid mass
DL-Arginine	C6 H14 N4 O2	Full match	Full match	No results
4-tert-Butylphenyl	C17 H18 O3	No match	Invalid mass	No results
α-Lactose	C12 H22 O11	No match	Invalid mass	Invalid mass
2,2,6,6-Tetramethy	C9 H19 N O	Full match	Full match	No results
BMK methyl glycid	C11 H12 O3	Full match	Full match	No results
BMK methyl glycid	C11 H12 O3	Full match	Full match	No results
Pantothenic acid	C9 H17 N O5	Full match	Full match	Full match
(3S)-3-(5-Methoxy-	C19 H20 N4 O2	No match	Invalid mass	No results
L-Glutamic acid	C5 H9 N O4	No results	Invalid mass	Invalid mass
6-Chloro-N2,N3-di	C12 H15 Cl N4	No match	Invalid mass	No results
(3S)-3-(5-Methoxy-	C19 H20 N4 O2	No match	Invalid mass	No results
Nipecotic acid	C6 H11 N O2	Full match	Full match	Full match
Succinic acid	C4 H6 O4	No results	Invalid mass	Invalid mass
JWH 018 adamanty	C24 H32 N2 O	Unused	Invalid mass	No results
Ambrosic acid	C15 H20 O4	No match	Invalid mass	No results
6-Hydroxycaproic a	C6 H12 O3	No results	Invalid mass	No results
1,8-Diazabicyclo [5	C9 H16 N2	Full match	Full match	No results
Caffeic acid	C9 H8 O4	Full match	Full match	Full match
1-Ethyl-3-[(1S,2S,3	C14 H26 N4 O4	Full match	Full match	No results
6-Benzyl-2-(tert-bu	C19 H20 N4 O2	No match	Invalid mass	No results
6-Benzyl-2-(tert-bu	C19 H20 N4 O2	No match	Invalid mass	No results
N-({(2R,4S,5R)-5-[1	C23 H28 N4 O2 S	No match	Invalid mass	No results
Leucylproline	C11 H20 N2 O3	Full match	Full match	Full match
(3R,4S)-4,6,8-Trihy	C11 H12 O6	No match	Invalid mass	No results
1,5-Naphthalenedi	C10 H10 N2	No match	Invalid mass	No results
Avermectin B1a	C48 H72 O14	No match	Invalid mass	No results
Oxyphenbutazone	C19 H20 N2 O3	No match	Invalid mass	No results
N1-[5-(tert-Butyl)-1	C16 H21 N O S	No match	Invalid mass	No results
1-Ethyl-3-[(1S,2S,3	C14 H26 N4 O4	Full match	Full match	No results

4-Acetoxy DiPT	C18 H26 N2 O2	No match	Invalid mass	No results
Hypoxanthine	C5 H4 N4 O	No results	Invalid mass	Invalid mass
8-(4-Sulfophenyl) c	C14 H20 O5 S	Unused	Invalid mass	No results
5-[(2-Hydroxy-2-ph	C19 H21 N O4	No match	Invalid mass	No results
Kynurenic acid	C10 H7 N O3	Full match	Full match	Full match
5-Fluoro CUMYL-P7	C22 H26 F N3 O	No match	Invalid mass	No results
{5-[({3-[(5-{[Benzyl(	C22 H27 N3 O4	No match	Invalid mass	No results
3-Succinoylpyridir	C9 H9 N O3	No match	Invalid mass	No results
3-[Hydroxy(phenyl	C16 H18 N2 O3	No results	Full match	No results
Lariciresinol 4-O-gl	C26 H34 O11	No match	Invalid mass	No results
3-Succinoylpyridir	C9 H9 N O3	Full match	Full match	No results
Chlortoluron	C10 H13 Cl N2 O	No match	Invalid mass	No results
2-Amino-4-methyl	C5 H7 N3	Full match	Full match	No results
3-Succinoylpyridir	C9 H9 N O3	Full match	Full match	No results
1,5-Naphthalenedi	C10 H10 N2	No results	Full match	No results
Rupatadine	C26 H26 Cl N3	No match	Invalid mass	No results
N-({(1S,4S,6S)-4-[2-	C27 H39 N3 O4	No match	Invalid mass	No results
4-(4-{5-[(2S)-1-Met	C16 H21 N5 O2	Full match	Full match	No results
1-{[(2R,3S,4R,5S)-3	C22 H34 N4 O6	No match	Invalid mass	No results
N-{[(2R,3S,4R,5S)-3	C22 H34 N4 O5	No match	Invalid mass	No results
3-[2-(1,3-Benzodio	C25 H28 O11	No match	Invalid mass	No results
7-Hydroxycoumari	C9 H6 O3	Full match	Full match	No results
D-(-)-Fructose	C6 H12 O6	Full match	Full match	Not the top hit
Ketotifen	C19 H19 N O S	No match	Invalid mass	No results
N1-(4-Fluoro-5-mo	C12 H14 F N3 O4	No match	Invalid mass	No match
Hexanoylglycine	C8 H15 N O3	Full match	Full match	Partial match
7,8-Bis(hydroxyme	C15 H22 O3	No match	Invalid mass	No results
3-Ureidopropionic	C4 H8 N2 O3	No results	Invalid mass	Invalid mass
Triethyl phosphate	C6 H15 O4 P	Full match	Full match	No results
Indole-3-acetyl-L-a	C14 H14 N2 O5	Full match	Full match	Full match
Hexanoylglycine	C8 H15 N O3	Full match	Full match	Partial match
N-Isovalerylglycine	C7 H13 N O3	Full match	Full match	Partial match
3-[2-(1,3-Benzodio	C25 H28 O11	No match	Invalid mass	No results
6-Deoxyhexopyran	C48 H74 O19	No match	Full match	No results
Azobenzene	C12 H10 N2	Full match	Full match	No results
3-Ethyl-4-hydroxy-	C17 H15 N O2	No match	Invalid mass	No results
1-{2-[4-(Diethylam	C20 H21 N3 O	No match	Invalid mass	No results
4-Methylumbellife	C10 H8 O3	Full match	Full match	No results
4-Oxo-6-(3-pyridyl	C10 H6 N4 O S	No match	Invalid mass	No results
4-Methylumbellife	C10 H8 O3	Full match	Full match	No results
N-[1,1'-Biphenyl]-4	C14 H13 N O	No match	Invalid mass	No results
N,N-Dimethylsphir	C20 H41 N O2	No match	Invalid mass	No results
4-Methylumbellife	C10 H8 O3	Full match	Full match	No results
3-(2-Hydroxyethyl)	C10 H11 N O	Full match	Full match	No results
PMK ethyl glycidat	C13 H14 O5	Full match	Full match	No results
4-Methylumbellife	C10 H8 O3	Full match	Full match	No results
PMK ethyl glycidat	C13 H14 O5	Full match	Full match	No results
7,8-Bis(hydroxyme	C15 H22 O3	Not the top hit	Full match	No results
[(3R,4S)-1-Acetyl-3	C21 H30 F N3 O3	No match	Invalid mass	No results
Pyridoxamine	C8 H12 N2 O2	Full match	Full match	No results
FUB-144	C23 H24 F N O	No match	Invalid mass	No results
4-Methylbenzophe	C14 H12 O	No results	Full match	No results

Estriol	C18 H24 O3	No match	Invalid mass	No results
5-Fluoro CUMYL-P7	C22 H26 F N3 O	No match	Invalid mass	No results
ADBICA	C20 H29 N3 O2	No match	Invalid mass	No results
Alprazolam	C17 H13 Cl N4	No match	Invalid mass	No results

Annotation Source: ChemSpider Search	Annotation Source: MassList Match	FISh Coverage	Molecular Weight	RT [min]
No results	Full match	80	314.2459	16.883
No results	Full match	65	256.07302	9.858
No results	Full match	43.75	188.10429	11.885
No results	Full match	76.19	196.05778	0.864
No results	Full match	30.23	166.0623	9.106
No results	Full match	33.33	268.074	12.484
No results	No results	26.09	155.0686	0.833
No results	Full match	23.81	434.12208	10.672
No results	No results	69.23	328.22557	14.49
No results	No match	11.54	210.07338	0.92
No results	Full match		192.02644	1.489
No results	Full match		137.04769	0.972
No results	No match		164.04742	1.624
No results	Full match		192.02641	1.078
No results	No results		210.13655	9.229
No results	Full match		390.27634	21.02
No results	No results		448.23026	7.086
No results	Full match		165.07898	3.7
No results	Full match		286.1625	1.63
No results	Full match		516.12758	11.078
No results	Full match		430.12569	11.502
No results	Full match		283.28722	20.932
No results	Full match		354.0952	7.396
No results	Full match		354.09537	5.637
No results	Full match		300.06387	11.667
No results	Full match		152.0472	8.317
No results	No results		216.08956	6.927
No results	Full match		278.15145	16.992
No results	Full match		244.12085	10.062
No results	Full match		174.08859	9.989
No results	Full match		187.06326	5.592
No results	No match		230.13034	16.656
No results	Full match		292.20336	14.499
No results	No match		230.13034	15.264
No results	Full match		206.05774	12.81
No results	Full match		268.0733	11.479
No results	Full match		147.05312	1.024
No results	Full match		162.06796	8.79
No results	Full match		294.21916	14.978
No results	Full match		194.09415	15.257
No results	Full match		370.30768	21.11
No results	Full match		426.3706	22.512
No results	No results		134.02286	2.658
No results	Full match		255.25591	19.869
No results	No match		296.23461	16.889
No results	No results		157.08512	0.836
No results	Full match		294.21916	16.471

No results	Full match		174.11167	1.15
No results	Full match		264.136	8.83
No results	No results		197.09001	1.012
No results	Full match		123.03224	1.256
No results	No results		350.20632	14.42
No results	Full match		290.12249	0.976
No results	Full match		296.23564	17.766
No results	Full match		250.15648	7.09
No results	No results		350.22291	16.891
No results	Full match		248.1409	15.233
No results	Full match		292.20342	17.176
No results	Full match		278.22408	17.8
No results	Full match		330.24098	14.983
No results	Full match		218.16689	13.414
No results	Full match		244.06957	1.653
No results	No match		115.02702	1.029
No results	Full match		202.12021	12.913
No results	Full match		246.1006	9.466
No results	Full match		179.09441	2.755
No results	Full match		246.10057	9.761
No results	No results		168.06868	7.761
No results	No results		288.13207	1.591
No results	Full match		193.1102	11.799
No results	No results		288.13207	1.951
No results	Full match		300.06365	13.024
No results	Full match		204.08964	5.647
No results	Full match		166.0623	6.53
No results	Full match		516.12776	8.229
No results	No results		494.23714	7.088
No results	No results		494.23714	6.408
No results	No results		242.12666	8.173
No results	No match		230.13034	11.681
No results	No results		462.24766	10.235
No results	No results		269.04509	10.992
No results	No results		228.15114	18.033
No results	No results		242.12664	8.603
No results	No results		311.19554	5.727
No results	Full match		194.10558	5.887
No results	No results		308.14762	10.016
No results	Full match		266.15224	15.57
No results	Full match		148.08869	15.709
No results	Full match		342.11631	0.936
No results	No match		166.04695	0.867
No results	No results		428.19005	9.252
No results	No match		230.11548	12.189
No results	No results		307.21059	0.673
No results	No results		380.21794	16.456
No results	Full match		192.0628	7.355
No results	Full match		202.12021	10.523
No results	Full match		232.15718	7.625
No results	Full match		205.0739	6.664
No results	Full match		180.04206	16.988

No results	Full match		165.07898	8.01
No results	No results		330.1792	12.526
No results	No results		312.24401	16.651
No results	No results		230.10144	0.979
No results	No match		340.13063	11.136
No results	No match		190.08368	9.539
No results	Full match		176.06787	7.112
No results	No results		452.32839	16.153
No results	Full match		182.05791	8.808
No results	Not the top hit		174.01569	1.074
No results	No results		512.21138	11.332
No results	No results		550.17512	1.12
No results	No results		411.19029	10.134
No results	No results		442.20567	11.121
No results	Full match		312.2304	16.457
No results	Full match		368.11089	8.913
No results	No results		534.13813	10.391
No results	No match		277.11614	0.99
No results	No results		208.03704	8.426
No results	Full match		166.0629	13.76
No results	Full match		122.04824	1.3
No results	No match		185.1165	4.536
No results	No results		302.12255	0.977
No results	Full match		278.22408	16.866
No results	Full match		166.0629	11.599
No results	No results		476.15407	7.102
No results	Full match		179.09441	8.857
No results	No results		260.12614	10.738
No results	Full match		165.07825	4.31
No results	No results		198.10047	4.307
No results	Full match		312.23024	16.046
No results	Full match		179.07931	1.002
No results	No results		362.20474	10.085
No results	Full match		178.06288	9.962
No results	No results		284.14855	5.144
No results	No results		379.14911	6.269
No results	No results		388.12196	0.939
No results	No results		550.17514	0.927
No results	No results		308.07939	12.68
No results	No results		377.20557	9.846
No results	No results		226.06865	0.951
No results	No results		295.0587	0.852
No results	No results		324.14265	8.049
No results	No results		377.20559	11.49
No results	Full match		122.03691	13.751
No results	Full match		370.21984	10.011
No results	Full match		148.08869	16.509
No results	Full match		282.14714	8.835
No results	No results		452.33566	9.907
No results	No results		378.16495	5.478
No results	No match		376.13779	8.644
No results	No results		378.19981	10.737

No results	No results		453.22605	8.94
No results	No results		213.0789	8.04
No results	No results		432.20015	8.702
No results	No results		232.10968	7.154
No results	No results		349.17442	7.444
No results	No results		230.10546	7.309
No results	No results		373.17452	8.233
No results	No results		418.11186	7.401
No results	Full match		282.14713	15.077
No results	Full match		266.15224	14.813
No results	Full match		282.14712	14.75
No results	Full match		266.15587	27.2
No results	Full match		240.06351	7.608
No results	Full match		226.09532	4.855
No results	Full match		162.03148	16.987
No results	No results		286.06959	6.206
No results	No results		298.11671	5.967
No results	No results		462.13835	5.845
No results	Full match		159.0684	2.333
No results	No match		446.12056	9.741
No results	No results		134.02041	0.986
No results	Full match		192.0786	9.032
No results	No results		132.07767	8.478
No results	Full match		174.11113	0.785
No results	No match		270.12285	15.261
No results	No results		359.14241	1.076
No results	Full match		157.14664	14.015
No results	Full match		192.0786	7.78
No results	Full match		192.0786	9.599
No results	Full match		219.11071	5.077
No results	No results		318.15369	0.86
No results	No results		147.0522	0.843
No results	No results		250.0943	8.445
No results	No results		318.15367	1.14
No results	Full match		129.07906	1.034
No results	No results		118.0255	1.784
No results	No results		386.23136	13.045
No results	No results		286.1165	2.805
No results	No results		132.07767	8.862
No results	No results		152.13117	7.483
No results	Full match		180.04193	7.594
No results	No results		314.19527	7.251
No results	No results		336.16399	1.144
No results	No results		336.164	0.878
No results	No results		470.20067	9.511
No results	Full match		228.14734	6.133
No results	No match		222.05184	9.381
No results	No results		141.05785	5.593
No results	No results		872.47859	16.752
No results	No results		324.14265	6.753
No results	No results		275.13692	2.263
No results	No results		314.1953	8.025

No results	No results		302.19522	7.788
No results	No results		136.03612	0.876
No results	No results		300.0994	12.209
No results	No results		327.14266	4.789
No results	Full match		189.04241	7.403
No results	No results		367.19896	7.682
No results	No results		419.17839	8.275
No results	No match		162.03171	0.567
No results	Full match		286.13141	7.937
No results	No results		568.21657	9.919
No results	Full match		179.05821	0.128
No results	No results		195.04094	0.804
No results	No results		109.0644	0.831
No results	Full match		179.05826	6.656
No results	No results		158.0844	6.579
No results	No results		415.18401	4.471
No results	No match		515.29209	18.111
No results	No results		315.16975	5.41
No results	No results		496.25322	6.888
No results	No results		480.25783	7.988
No results	No results		550.16956	9.913
No results	Full match		162.03148	7.413
No results	Full match		180.06268	0.95
No results	No results		309.12093	4.297
No results	No match		283.09167	2.682
No results	Full match		173.10461	8.322
No results	No results		250.15276	0.706
No results	No results		132.05241	0.831
No results	Full match		182.07048	10.973
No results	Full match		290.09001	9.441
No results	Full match		173.10461	8.558
No results	Full match		159.08877	6.238
No results	No results		550.16902	11.433
No results	No results		954.48408	17.46
No results	No results		182.08415	8.479
No results	No results		265.10628	5.815
No results	No results		319.16429	6.52
No results	Full match		176.04732	8.593
No results	No results		230.02764	0.699
No results	Full match		176.04732	10.094
No results	No match		211.09573	2.501
No results	No results		309.3026	21.125
No results	Full match		176.04732	8.968
No results	Full match		161.08389	8.856
No results	Full match		250.08475	0.376
No results	Full match		176.04732	9.592
No results	Full match		250.08455	29.608
No results	Full match		250.1565	0.386
No results	No results		396.17767	11.686
No results	Full match		168.08991	4.416
No results	No results		349.18825	8.499
No results	Full match		196.08869	16.273

No results	No match		271.14156	10.48
No results	No results		367.19896	8.165
No results	No match		326.1935	8.058
No results	No results		308.07939	12.913

# ChemSpider Results	# mzCloud Results	# mzVault Results	mzCloud Best Match	Area: CD015.raw (F1072)
0	4	0	100	15924477.84
0	6	2	100	10828112.04
0	2	2	100	4637145.5
0	3	2	100	62864899.4
0	16	12	100	533197.262
0	1	1	100	17714863.02
0	1	1	100	1599231.95
0	2	0	100	309380.7674
0	1	0	100	28001794.01
0	2	2	99.4	17321018.59
0	4	2	98.9	292079746.9
0	18	11	98.7	21146047.98
0	4	4	98.4	12478442.97
0	2	1	98.2	166913784
0	2	0	97.8	5455987.852
0	10	8	94.7	3777008.66
0	2	0	94.3	2435865.88
0	1	4	93.4	41978412.56
0	3	2	91.5	192941
0	1	0	91.4	1056089.652
0	2	0	89.7	21193995.71
0	2	0	89.4	1824945.322
0	2	1	88.6	20832052.52
0	2	1	88.6	2513696.349
0	2	0	87.7	1478841.179
0	3	5	87.2	3016976.131
0	1	0	87	1479469.785
0	6	6	86.7	32462608.12
0	2	0	86.4	872295.3707
0	2	2	86.1	1244561.62
0	4	8	83.9	67903301.84
0	1	0	83.9	4901540.749
0	4	0	83.9	6658694.594
0	2	0	83.8	29526225.82
0	4	2	83.5	1656596.468
0	1	1	83.3	1524844.877
0	1	1	83.3	6197893.851
0	1	0	81.8	1747793.571
0	4	2	81.3	17230599.1
0	2	0	81.1	768852.1122
0	1	1	80.7	1508423.145
0	2	2	80.7	3367412.396
0	2	2	80.7	13752384.6
0	2	2	80.3	2877493.99
0	2	0	80	3277730.407
0	4	4	79.4	422419680.3
0	4	2	78.8	8397721.789

0	4	4	78.8	50904158.74
0	6	2	77.2	940643.7871
0	1	1	77.2	11375139.59
0	2	2	77.1	6433316.696
0	4	0	75.9	1407816.253
0	3	0	75.5	7244534.529
0	6	0	74.9	3322794.918
0	2	0	74.2	3071341.491
0	2	0	73.5	480337.2344
0	5	0	73.2	5422805.483
0	2	0	73.2	1597591.616
0	3	0	72.5	4052085.171
0	1	0	72.3	100076524.1
0	3	2	72.1	143741.0077
0	6	2	70.8	2541261.949
0	1	1	70.6	33319447.92
0	1	1	70.4	1431938.684
0	4	2	69.3	1651776.11
0	10	0	69.1	6053521.506
0	2	1	69.1	957303.3369
0	1	0	67.7	454882.316
0	2	0	67.4	3254888.124
0	3	1	67.1	463846.4374
0	4	0	67.1	4648247.796
0	2	0	67	690177.0646
0	2	2	67	510262.0503
0	8	7	67	4482243.649
0	1	0	66.9	619631.1753
0	2	0	66.1	9157353.235
0	2	0	65.7	967115.093
0	1	0	64.7	1861994.181
0	1	0	64.7	524267.4853
0	3	0	64.6	1228799.861
0	1	0	64.5	477188.1359
0	2	0	64.4	796157.5484
0	1	0	64.3	3721622.634
0	1	0	64.2	615020.2269
0	2	0	64.1	1624146.543
0	1	0	63.3	1542086.162
0	3	0	63.3	1172747.103
0	6	0	63.3	2624303.432
0	8	8	63.2	27198368.62
0	2	0	63.2	9180477.951
0	1	0	62.8	487897.4993
0	1	0	62.8	624888.0689
0	2	0	62.8	1490888.48
0	1	0	62.7	559298.6251
0	2	2	62.7	1766615.385
0	1	0	62.7	329834.6354
0	4	0	62.6	981380.3859
0	7	5	62.5	614373.3052
0	2	2	62.4	9393244.901

0	3	0	62.2	1488655.726
0	1	0	62.2	800721.9597
0	4	0	62	37502.10498
0	5	0	61.9	4612999.42
0	1	0	61.8	1283735.574
0	2	0	61.8	2251908.329
0	4	4	61.7	7494059.321
0	2	0	61.6	9567435.733
0	3	1	61.6	1623665.543
0	2	2	61.5	4397801.529
0	1	0	61.3	716531.6985
0	1	0	61.1	1131506.64
0	6	0	61.1	986511.0068
0	2	0	61	7758089.709
0	2	0	60.9	28620254.76
0	1	0	60.6	1745819.566
0	1	0	60.5	2639407.836
0	1	0	60.4	22683528.98
0	1	0	60.4	662301.5344
0	1	0	60.3	1471251.757
0	2	2	60.2	9495008.661
0	2	0	60.2	1373180.205
0	2	0	60.2	8039982.423
0	3	0	60.1	3337715.469
0	2	0	60.1	704470.9936
0	1	0	59.7	94364.4515
0	4	1	59.7	23376029.61
0	1	0	59.6	367895.8308
0	1	1	59.5	493235.2455
0	4	0	59.4	7637553.156
0	1	0	59.4	6458043.944
0	1	1	59.4	18815043.14
0	1	0	59.2	764188.6307
0	2	0	59.1	1391744.612
0	2	0	59	1514279.337
0	6	0	58.9	570905.1659
0	4	4	58.7	65159788.29
0	1	0	58.7	5677268.867
0	2	0	58.6	321855.8516
0	5	0	58.6	1091322.094
0	2	2	58.5	65440290.92
0	1	0	58.3	4742367.373
0	2	0	58.2	1151142.734
0	4	0	58.2	372470.4603
0	1	1	58.1	475908.0414
0	1	0	58	569577.208
0	2	0	57.9	1503465.781
0	2	0	57.5	417343.7113
0	1	0	57.4	4590551.358
0	6	0	57.4	1928045.248
0	1	1	57	594455.9153
0	2	0	57	1811922.713

0	2	0	56.9	13298615.54
0	3	0	56.8	716452.8224
0	1	0	56.8	521295.9133
0	2	0	56.7	292675.4829
0	2	0	56.7	1695409.905
0	6	0	56.5	1242257.014
0	1	0	56.4	621841.0695
0	2	0	56.2	1225361.029
0	4	0	56.1	22033921.92
0	3	0	55.8	1000580.325
0	2	0	55.1	3205363.265
0	6	2	55.1	380810.716
0	1	0	53.8	11962048.24
0	1	0	53.5	1845275.43
0	4	0	53	12880792.54
0	1	0	53	667346.2309
0	1	0	52.9	1023829.492
0	1	0	51.2	684314.896
0	1	0	50.2	3939226.474
0	2	2	48.5	41921117.47
0	4	6	48.1	74387239.9
0	1	0	46.8	2032580.213
0	2	4	46.8	876574.3665
0	1	0	46.3	3437657.861
0	1	0	46.3	573180.7734
0	1	1	45.8	7417414.687
0	2	0	45.5	642495.5206
0	1	0	45.4	1068752.77
0	3	0	45.3	2440863.193
0	2	1	45.2	2467707.152
0	3	0	45.1	10088278.83
0	4	2	45	1987260.893
0	1	0	44.8	309166.1716
0	3	0	44.7	1824545.903
0	2	8	44.3	24117917.27
0	4	4	44.2	6354626.906
0	2	0	44.1	3855356.357
0	1	0	43.9	1670067.542
0	1	0	43.9	370417.9767
0	1	0	43.9	600437.9809
0	2	1	43.7	1915135.03
0	1	0	43.6	1245556.367
0	3	0	43.6	12328968.54
0	3	0	43.6	63715943.58
0	1	0	43.5	908695.4282
0	1	1	43.4	2112153.338
0	1	0	43.2	158864.2207
0	1	0	43	1244822.665
0	1	0	43	10253545.65
0	1	0	42.8	1092211.028
0	2	0	42.7	8522803.592
0	2	0	42.6	1515359.286

0	2	0	42.4	1062039.797
0	2	1	42.4	3459268.341
0	1	0	42.3	4754219.868
0	2	0	42.2	12234.37588
0	1	1	42.2	194175.5515
0	2	0	42.2	2060022.223
0	2	0	42.1	1366863.827
0	2	0	42.1	695276.7923
0	2	0	42	441563.7553
0	1	0	42	1124427.024
0	3	0	42	1355594.187
0	1	0	41.9	10947830.83
0	1	0	41.7	362583.6258
0	2	0	41.7	1878545.198
0	1	0	41.6	362806.2817
0	2	0	41.6	2113266.239
0	2	0	41.5	980314.0236
0	1	0	41.5	859293.5671
0	1	0	41.3	388620.3055
0	1	0	41.2	344001.6692
0	1	0	41.2	2845951.491
0	4	0	41.1	5264571.848
0	4	4	41.1	151811163.1
0	2	0	40.9	2301282.197
0	1	1	40.9	16826709.5
0	1	3	40.8	156426.3688
0	1	0	40.8	5558983.113
0	1	1	40.8	12362525.24
0	1	0	40.8	70430.94779
0	1	1	40.8	1337079.743
0	1	3	40.6	1450008.046
0	1	2	40.6	418873.2174
0	1	0	40.6	727350.2453
0	1	0	40.6	7633151.891
0	1	0	40.3	181782.1847
0	1	0	40.3	1401604.331
0	2	0	40.2	457548.7199
0	1	0	40	282407.5422
0	1	0	40	4805514.034
0	1	0	39.7	2061789.258
0	2	0	39.7	4684911.986
0	1	0	39.5	521510.7942
0	1	0	39.5	1492873.752
0	2	0	39.4	8461794.745
0	2	0	39.4	53574.93432
0	1	0	39.2	577842.0821
0	2	0	39.2	64355.42103
0	1	0	39.1	43019.08469
0	2	0	39.1	12686288.82
0	1	0	39	2273891.282
0	2	0	38.7	21377393.1
0	3	0	38.6	3707778.339

0	2	0	38.3	15987258.09
0	1	0	38	4162134.1
0	1	0	37.9	4648699.126
0	1	0	37.8	230502.5803
